# Pharmacodynamic modelling of resistance to epidermal growth factor receptor inhibition in brain metastasis mouse models

**DOI:** 10.1007/s00280-018-3630-8

**Published:** 2018-07-27

**Authors:** Emma C. Martin, Leon Aarons, James W. T. Yates

**Affiliations:** 1 Centre for Applied Pharmacokinetic Research, Manchester Pharmacy School, The University of Manchester, Manchester, UK; 2 DMPK, Oncology, Innovative Medicines and Early Development, AstraZeneca, Cambridge, UK

**Keywords:** Bioluminescence, Resistance, Brain metastasis, Xenograft, EGFR inhibitor

## Abstract

**Purpose:**

Epidermal growth factor receptor (EGFR) is thought to play a role in the regulation of cell proliferation; with its activation stimulating tumour growth. EGFR inhibitors have shown promise in the treatment of cancer, particularly in non-small cell lung cancer, however, resistance is observed in the majority of patients. A tumour growth model was developed aiming to explain this resistance.

**Methods:**

The model incorporating populations of both sensitive and resistant cells were fitted to data from a study of EGFR inhibitor AZD3759 in brain metastasis mouse models. The observed regrowth of tumours in higher dose groups suggested the development of resistance to treatment. The bioluminescence observations were highly variable, covering many orders of magnitude, so to assess how reliable the model was, the parameter estimates were compared to those found in less noisy subcutaneous mouse models.

**Results:**

The fitted model suggested that resistance was mainly due to a proportion of cells being resistant at baseline, and the contribution of mutations occurring during the study leading to resistance was negligible. Estimated growth rate and dose–response was found to be comparable between brain metastasis and subcutaneous mouse models.

**Conclusions:**

The developed model to describe resistance suggests that the resistance to EGFR-inhibition seen in these xenografts is best described by assuming a small percentage of cells are resistant to treatment at baseline. This model suggests changes to dosing and dosing schedule may not prevent resistance to treatment developing, and that additional treatments would need to be used in combination to overcome resistance.

## Introduction

The targeting of epidermal growth factor receptor (EGFR), a cell-surface receptor in the ErbB family, has shown anti-tumour activity and has been seen as a promising therapy in oncology [1]. There are different classes of EGFR inhibitors, small molecules, such as gefitinib and erlotinib, that inhibit the tyrosine kinase inhibitors (TKIs) as well as monoclonal antibodies (mAbs) such as cetuximab and panitumumab [2, 3]. EGFR is thought to play a role in the regulation of cell proliferation [4], its activation stimulates tumour growth and progression, promotes proliferation, angiogenesis, invasion, metastasis and inhibition of apoptosis [1].

Despite the early promise of EGFR-inhibition in the treatment of cancer, the development of resistance to treatment is observed in the majority of patients [3, 5, 6]. The mechanisms of resistance are not fully understood, but a number have been proposed, such as secondary mutations, activation of alternate signalling, impairment of the apoptosis pathway, histological transformation [3, 5, 7–10] and brain metastasis that are protected from treatment by the blood–brain barrier [11]. Resistance can more broadly be described as being due to pre-existing resistant cells or de novo evolution [12–14].

A mathematical model to describe resistance to EGFR inhibitors has previously been developed in cell lines, describing two populations of cells, sensitive and resistant, which were found to exhibit different growth kinetics. The model was used to optimise the dosing schedule to prevent resistance [15]. A further model assuming acquired resistance has been fitted to both gefitinib and erlotinib in patient-derived xenografts, however, the possibility that a fraction of the cells are resistant at baseline was not considered [16]. A general framework has been proposed to model resistance to EGFR-inhibition, when used in combination with a cytotoxic treatment. The framework again suggests describing separate populations of sensitive and resistant cells, which both exhibit exponential growth, although the model was not fitted to experimental data [17].

Here we present a simple mathematical model to describe the developed resistance to treatment with EGFR inhibitors, which can then be used to assess the impact of different treatment regimens. The model was tested on data arising from a complex brain metastasis mouse model following treatment with AZD3759, an EGFR inhibitor designed to effectively cross the blood–brain barrier (BBB). The estimated dose–response curve developed in the brain metastasis mouse model was then compared to the dose–response estimated from standard subcutaneous xenograft mouse models.

## Materials and methods

Pharmacokinetic (PK) and tumour growth inhibition data were available from two preclinical studies investigating the use of EGFR inhibitor AZD3759, which is ATP competitive, similar to gefitinib. However, is has been designed to cross the BBB to tackle central nervous system (CNS) metastasis in patients with activating mutations of EGFR in non-small cell lung cancer (NSCLC) [18, 19]. AZD3759 is now being tested in early clinical trials. One preclinical study was carried out in subcutaneous xenografted mice and the other in brain metastasis mouse models.

The study in brain metastasis models had longer follow-up and resistance was observed within several weeks. A model of resistance to treatment with EGFR inhibitors was developed using this dataset. Due to the highly variable nature of the bioluminescence observations in the brain metastasis models, the estimated model parameters were compared to those found in the less variable subcutaneous mouse models to assess reliability.

### Subcutaneous xenograft mouse models

Data were available from a study in subcutaneous xenografted mice, where AZD3759 was tested at four dose levels (3.75, 7.5, 15 and 30 mg/kg dosed daily), a clear dose–response was observed. Approximately 15 mm^3^ of cells were initially implanted, tumour size at the start of dosing was approximately 180 mm^3^, then measured roughly every 4 days using callipers, tumour volume was then calculated as *V* = (length × width^2^)/2. In total, 38 animals were included in the study, 7 control animals, and 7 or 8 animals in each dose group. A number of tumour growth models were tested but due to the relatively short follow-up time of 15 days and no resistance to AZD3759 treatment or slowing of growth due to competition between cells for resources such as oxygen [20, 21] being observed, a simple exponential growth model was found to sufficiently describe the data. The dose–response was assumed to be proportional to the tumour volume, both linear and *E*_max_ dose–response models (Eq. 1) were tested, with an *E*_max_ model found to better describe the data. Treatment was assumed to cause cell death rather than causing a decrease in the proliferation rate, in line with [22], as reductions in tumour size from baseline are observed. The response to treatment was assumed to remain constant throughout the study as dosing was daily.

The model described by Eq. (1) was fitted to the data using the first-order conditional estimation method (FOCE with interaction) in NONMEM 7.3 [23], inter-individual variation (IIV) and residual error were assumed to be log-normally distributed. The magnitude of IIV was expressed as a coefficient of variation (CV%), the square root of the variance. IIV was initially assumed on all parameters, but was not included in the final model if there were difficulties in estimation or if it was estimated to be small:

1 $$\frac{{{\text{d}}V}}{{{\text{d}}t}}=\lambda V - ~\frac{{{E_{{\text{max}} }}~{D^\gamma }}}{{{\text{ED}}_{{50}}^{\gamma }+~{D^\gamma }}}V,$$

where $V$ is the volume of the tumour, ${E_{{\text{max}} }}$ is the maximum effect of treatment, $D$ is the dose and ${\text{E}}{{\text{D}}_{50}}$ is the dose at which 50% of the maximum effect is achieved. Visual predictive checks (VPCs) were used to assess the model fit, 1000 datasets were simulated from the model and 95% prediction intervals were calculated by taking the 2.5th and 97.5th centiles.

### Brain metastasis mouse models

**Fig. 1:**
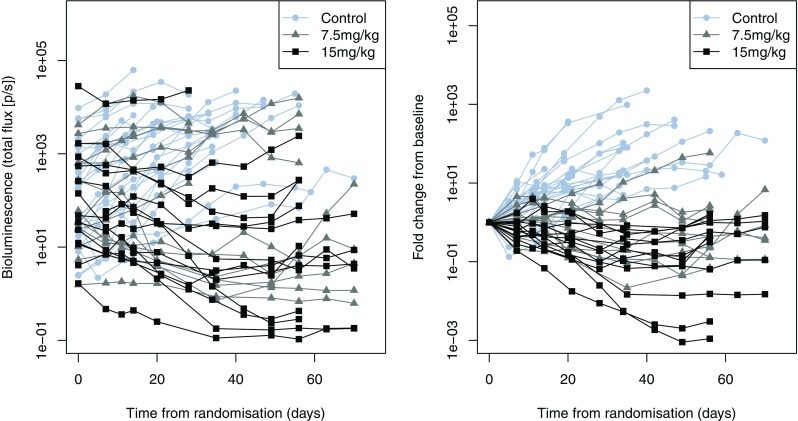
Raw bioluminescence measurements for brain metastasis data (left) with fold change from baseline (right)

Additional data were available in brain metastasis mouse models, where the human NSCLC cell line PC9 (exon 19 deletion) was transfected with the GL4.50[luc2/CMV/Hygro] vector containing the luciferase gene (PC9_Luc) using lipofectamine LTX. The cells were implanted into 51 immunodeficient nude mice, 25 controls and 13 in each dose group. Due to the location of the tumours bioluminescence imaging was used to quantify tumour burden, bioluminescence signals were measured using a Xenogen imaging system [24]. The bioluminescence data were extremely variable; both at baseline, which covered many orders of magnitude, and in growth rate (Fig. 1 left); however, a clear dose–response can be seen with two doses (7.5 and 15 mg/kg) when considering change from baseline (Fig. 1 right).

**Fig. 2:**
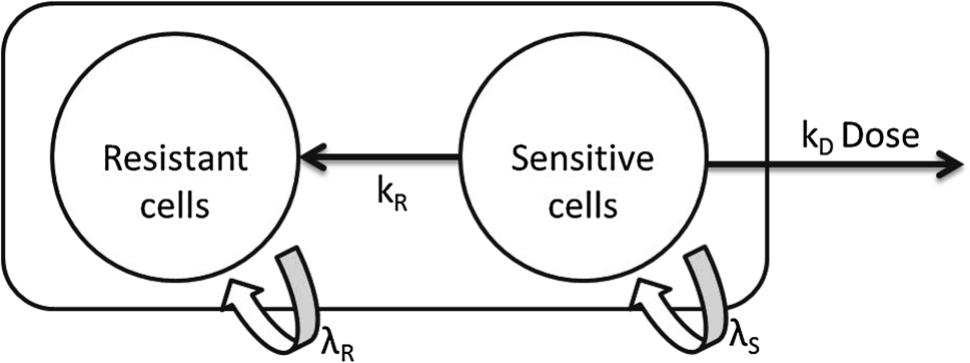
Structure of model for resistance to treatment, the total tumour volume consists of both sensitive and resistant cells with proliferation rates ${\lambda _{\text{S}}}$ and ${\lambda _{\text{R}}}$, rate constant ${k_{\text{R}}}$ describes the conversion of sensitive cells to resistant cells and ${k_{\text{D}}}$ describes cells death due to treatment

The follow-up time in this study was up to 70 days and evidence that tumours stopped responding to treatment was observed, particularly in the higher dose group where the tumours begin to regrow despite continuous daily dosing. Therefore, in the model, the tumour was assumed to be made up of two populations of cells, those sensitive to treatment and those which were resistant (Fig. 2). Growth was assumed to be exponential, as has previously been used in bioluminescence tumour growth data [25]. It was assumed that sensitive and resistant cells could both be described using separate exponential growth terms. In the brain metastasis data, it was not possible to fit an *E*_max_ model for dose–response, as only two doses were tested, so a linear model with cell death rate (${k_{\text{D}}}$) was assumed (response = ${k_{\text{D}}}D$, see Eq. 2).

The differential equations used to describe the resistance model are given in Eqs. (2–4). Two different hypotheses for the mechanism of how resistance occurs were considered.

- *Hypothesis 1* Due to heterogeneity within the tumour, a fraction of cells are resistant to treatment at baseline.
- *Hypothesis 2* The tumour is composed solely of sensitive cells and these can become mutated during the study leading to resistance [13, 14].

Both hypotheses were tested independently and then together. When cells were assumed to be resistant at baseline the initial conditions were: ${\text{Sensitive}}\,({\text{0}})=p\,{\text{Total}}\,{\text{(0)}}$ and ${\text{Resistant}}\,(0)=(1 - p)\,{\text{Total}}\,(0),$ where $p$ is the proportion of cells which are sensitive at baseline; and a logit transformation $(p=\exp (S)/(1+\exp (S))$ was used to constrain $p$ between 0 and 1. The treatment was assumed to act on sensitive cells only and, as in the subcutaneous model, was assumed to cause cell death. The treatment effect was assumed to be proportional to dose and the effect on sensitive cells was assumed to be constant over the course of the study:

2 $$\frac{{{\text{dSensitive}}~}}{{{\text{d}}t}}=~{\lambda _{\text{S}}}\,{\text{Sensitive}} - ~{k_{\text{R}}}\,{\text{Sensitive}} - ~{k_{\text{D}}}D\,{\text{Sensitive}},$$

3 $$\frac{{{\text{dResistant}}}}{{{\text{d}}t}}=~{\lambda _{\text{R}}}\,{\text{Resistant}}+~{k_{\text{R}}}\,{\text{Sensitive,}}$$

4 $${\text{Total}}={\text{Sensitive}}+{\text{Resistant.}}$$

The model was fitted to raw bioluminescence data using FOCE with interaction in NONMEM 7.3, inter-individual variation and residual error were again assumed to be log-normally distributed.

## Results

### Subcutaneous xenograft mouse models

**Fig. 3:**
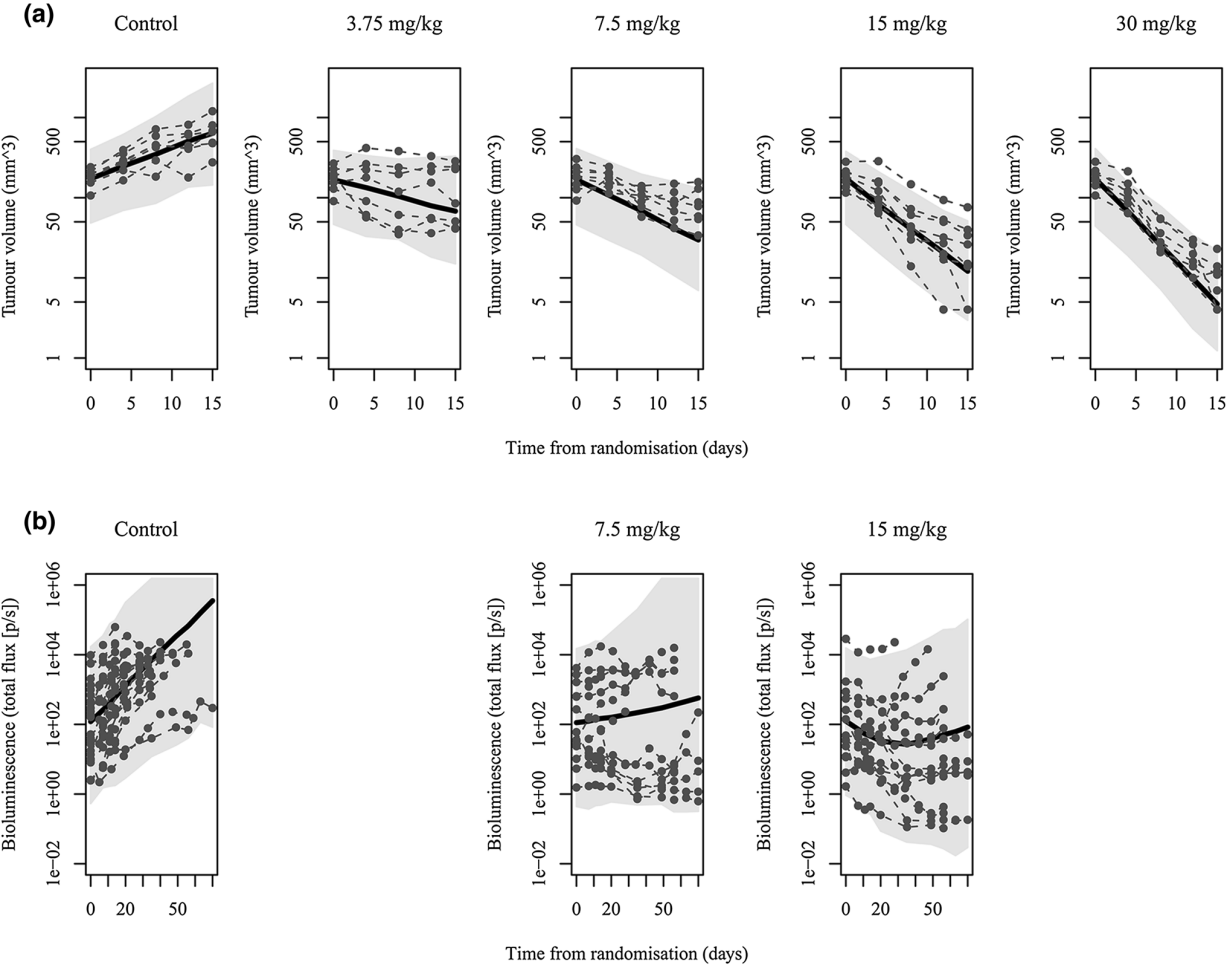
Visual predictive check for **a** the subcutaneous models and **b** brain metastasis models by dose, with population model fit (solid line), maximum tumour size (horizontal dashed line) and 95% prediction intervals (shaded)

The parameter estimates for the exponential growth model fitted to the subcutaneous xenografted mice are given in Table 1. The visual predictive checks in Fig. 3a as well as other diagnostic checks indicate that the model describes the dose–response well, and the inter-individual variation is well captured.

**Table 1:** Parameter estimates from exponential model fitted to the subcutaneous xenograft data, with relative standard errors from bootstrapping

Parameters	Estimate (RSE%)	IIV CV%
*λ* (proliferation rate constant, 1/day)	0.0855 (10.8)	34.9
*E*_max_ (mm^3^/day)	0.519 (27.8)	–
ED_50_ (mg/kg)	21.5 (83.2)	–
Gamma (–)	0.617 (45.2)	–
Baseline (mm^3^)	175 (5.5)	30.5
Residual error (%)	12.7	

### Brain metastasis mouse models

The resistance to treatment observed in the brain metastasis models was found to be best explained by hypothesis 1; assuming a proportion of cells were resistant at baseline. This hypothesis was preferred based on a number of factors including the AIC (4029.19 for hypothesis 1, 4028.59 for hypothesis 1 + 2) as well as the model fits and the relative impact of each mechanism of resistance on the absolute number of resistant cells. The model fit for hypothesis 2 alone was poor so was not considered. The contribution of sensitive cells mutating to become resistant during the study was estimated to be negligible, although this was with high relative standard error. The parameter estimates are given in Table 2.

**Table 2:** Parameter estimates from resistance model fitted to the bioluminescence data, with relative standard errors from bootstrapping

Parameters	Hypothesis 1		Hypothesis 1 + 2 combined	
	Estimate (RSE%)	IIV CV%	Estimate (RSE%)	IIV CV%
*λ*_S_ [sensitive cell proliferation, × 10^6^ (p/s)/day]	0.108 (8.2)	48.2	0.0992 (11.7)	48.9
*λ*_R_ [resistant cell proliferation, × 10^6^ (p/s)/day]	0.0556 (49.1)	–	0.0708 (49.3)	–
*k*_R_ (conversion rate, 1/day)	–	–	1.54 × 10^−6^ (139)	–
*k*_D_ (cell death rate, 1/day)	0.0139 (11.7)	11.0	0.0132 (19.0)	9.08
Baseline [× 10^6^ (p/s)]	137 (19.6)	245	177 (34.0)	248
Percentage sensitive at baseline (%)	98.7 (9.3)	48.4	99.6 (18.9)	135
Residual error (%)	19.0		16.5	

**Fig. 4:**
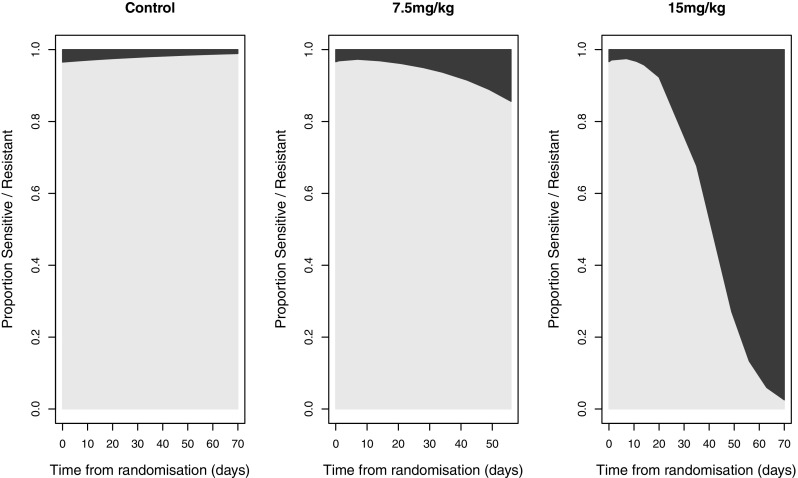
Estimated proportion of cells sensitive (light) and resistant (dark) to treatment over the course of the study by dose

The proportion of the tumours estimated to be resistant to treatment over the study by dose level is shown in Fig. 4. In the control group, the proportion of resistant cells decreases over the study as sensitive cells were estimated to proliferate more quickly, consistent with previous findings [14, 15]. In the two-treated groups, the proportion of resistant cells increases over the study, as the number of sensitive cells is reduced by the treatment, while the resistant cells continue to proliferate. In the 15 mg/kg dose group by the end of the study, nearly the entire tumour is estimated to be resistant to treatment, explaining the observed regrowth.

The visual predictive checks in Fig. 3b show that the model is able to describe the large variability in the data, which is mostly attributed to inter-individual variation at baseline. The resistance to treatment is well characterised by the model, including the description of the tumour regrowth observed in the 15 mg/kg dose group. In the control group, some tumour sizes are predicted to be higher than those observed due to the drop out of animals with the largest tumour burdens.

**Fig. 5:**
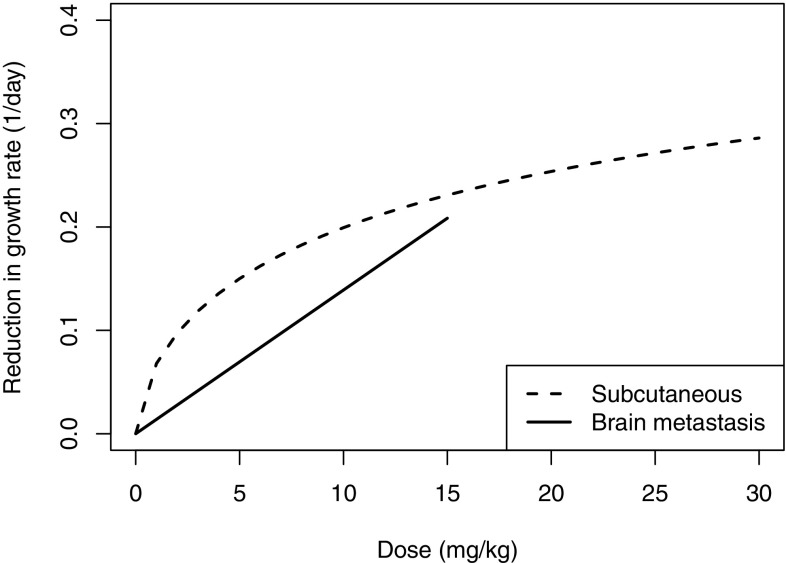
Dose–response curves for the subcutaneous and brain metastasis mouse models

The estimated growth rates in the two mouse models were very similar, approximately 0.09 in the subcutaneous model (Table 1) and 0.11 in the brain metastasis model (Table 2). PK data showed similar concentrations were achieved in the brain as in plasma, showing AZD3759 can pass through the BBB, enabling comparison of dose–response between the animal models. The dose–response curves (Fig. 5) were estimated to be similar in magnitude between the two mouse models, although the shape differs due to the number of doses tested. These similarities suggest that despite the high level of variability observed in the brain metastasis models, when using appropriate modelling techniques it is possible to reach a reliable estimate of the dose–response.

## Discussion

The model proposed describes AZD3759 effectiveness in both subcutaneous and brain metastasis in mouse models. The preferred model based on AIC and model fit showed that the resistance to EGFR-inhibition was due to a small number of cells (approximately 1%) being resistant to treatment at baseline; while the contribution from cells mutating to become resistant during the study was negligible. Consequently, this model suggests that changes to dose level or dosing schedule may not help to overcome resistance in this case. If treatment is continued long enough there will be very few sensitive cells remaining, while the resistant cells will continue to proliferate at the same rate, resulting in all treated tumours ultimately ending up being the same volume, regardless of the dose administered.

The selected hypothesis would suggest that additional treatments which are effective against cells resistant to EGFR inhibitors need to be used in combination with EGFR-inhibition to avoid resistance to treatment; as has previously been suggested [14, 17]. However as these conclusions are based solely on hypothesis selection, further work is needed to reinforce these conclusions experimentally; especially given the highly variable data and the modest difference in AIC.

The bioluminescence data are highly variable which could in part be due to a number of issues around the imaging itself. Studies have found that the estimated tumour size from bioluminescence imaging does not correlate with true tumour volume [25, 26]. Measurements can be affected by the location of the tumour and the positioning of the animals during imaging [25, 27, 28], non-uniformity of luciferase expression across tumour cells, quenching of light emissions by brain tissue, skin and bone [29], as well as the ability of luciferase to diffuse through the blood–brain barrier [30]. However, the agreement of the model parameter estimates with those estimated using the less variable subcutaneous mouse models may suggest the estimates of dose–response are reliable. In the brain metastasis models, there were a small number of animals that dropped out of the study early due to large tumour burdens. This was investigated using a joint modelling approach to account for dropout [31], but the relatively small number of dropouts (10% of all animals) was found not to significantly affect the estimation of parameters in the model. However, this drop out could explain the over-estimation of the tumour volume in the control and 7.5 mg/kg dose groups in the visual predictive check in Fig. 3.

The two mouse models give very similar estimates of growth rate and dose–response despite differences in baseline and variability. Subcutaneous models are simpler and more convenient than the more complex brain metastasis models; and therefore in some cases it may be appropriate to use subcutaneous xenograft models to assess dose–response. However, it must first be shown that the treatment is crossing the blood–brain barrier, reaching the site of action, as was determined to be the case with AZD3759 [19].

Simple mathematical models were used to describe the growth of both sensitive and resistant populations of tumour cells, but it should be possible to use more complex models, for example, to describe the slowing of the growth rate as the tumour burden increases, if further data were available. Further work could be done to model combinations of treatment, potentially utilising the framework suggested for describing the combination of EGFR inhibition with a cytotoxic treatment, describing populations of cells resistant to one or both treatments [17].
